# DNA Resection at Chromosome Breaks Promotes Genome Stability by Constraining Non-Allelic Homologous Recombination

**DOI:** 10.1371/journal.pgen.1002633

**Published:** 2012-03-29

**Authors:** Frederick J. Tan, Margaret L. Hoang, Douglas Koshland

**Affiliations:** 1 Howard Hughes Medical Institute and Department of Embryology, Carnegie Institution, Baltimore, Maryland, United States of America; 2 Department of Molecular and Cell Biology, University of California Berkeley, Berkeley, California, United States of America; 3 Department of Biology, Johns Hopkins University, Baltimore, Maryland, United States of America; The University of North Carolina at Chapel Hill, United States of America

## Abstract

DNA double-strand breaks impact genome stability by triggering many of the large-scale genome rearrangements associated with evolution and cancer. One of the first steps in repairing this damage is 5′→3′ resection beginning at the break site. Recently, tools have become available to study the consequences of not extensively resecting double-strand breaks. Here we examine the role of Sgs1- and Exo1-dependent resection on genome stability using a non-selective assay that we previously developed using diploid yeast. We find that *Saccharomyces cerevisiae* lacking Sgs1 and Exo1 retains a very efficient repair process that is highly mutagenic to genome structure. Specifically, 51% of cells lacking Sgs1 and Exo1 repair a double-strand break using repetitive sequences 12–48 kb distal from the initial break site, thereby generating a genome rearrangement. These Sgs1- and Exo1-independent rearrangements depend partially upon a Rad51-mediated homologous recombination pathway. Furthermore, without resection a robust cell cycle arrest is not activated, allowing a cell with a single double-strand break to divide before repair, potentially yielding multiple progeny each with a different rearrangement. This profusion of rearranged genomes suggests that cells tolerate any dangers associated with extensive resection to inhibit mutagenic pathways such as break-distal recombination. The activation of break-distal recipient repeats and amplification of broken chromosomes when resection is limited raise the possibility that genome regions that are difficult to resect may be hotspots for rearrangements. These results may also explain why mutations in resection machinery are associated with cancer.

## Author Summary

Chromosomes encode most of the genetic information necessary for cells to function. When large changes in chromosome structure occur, these changes can lead to a variety of diseases, including cancer. One type of DNA damage that triggers chromosomal changes is a DNA double-strand break. These breaks are often healed correctly by searching the cell for a second undamaged copy of the chromosome and using it as a repair template. However, when breaks occur near DNA sequences that are repeated tens to thousands of times in a genome, these breaks may be healed using a repeat on a different chromosome, leading to a translocation and resulting in the loss or gain of genetic information. In this study, we examine how the extensive processing that normally occurs at double-strand breaks affects the frequency of chromosome rearrangements in yeast. Unexpectedly, we find that limited processing of double-strand breaks leads to more, not fewer, chromosome rearrangements even when breaks occur far from repeated sequences. Furthermore, limited processing allows some cells to duplicate damaged chromosomes resulting in multiple rearrangements from just one break. We discuss possible mechanisms by which these repeats generate rearrangements, as well as how extensive processing of double-strand breaks prevents the accumulation of large-scale mutations.

## Introduction

Large-scale genome rearrangements such as deletions, duplications, and translocations contribute to evolution and cancer [1], [2]. Many of these rearrangements result from homologous recombination [3]–[5]. Homologous recombination occurs when a recipient sequence surrounding a DNA double-strand break is paired with a homologous donor sequence that acts as a repair template [6]. If recipient sequences contain only unique DNA, then donor sequences are limited to sister chromatids or homologs, and recombination will maintain genome structure. However, if recipient sequences contain elements of a dispersed repetitive DNA family, then potential donor sequences might occur anywhere in the genome, and recombination with these non-allelic donors can lead to genome rearrangements. Thus, how often recipient sequences contain repetitive DNA impacts the frequency of recombination-mediated genome rearrangements and consequently genome stability.

The probability that recipient sequences will contain repetitive DNA depends in part upon 5′→3′ DNA resection machinery [7], [8]. This resection machinery requires Sgs1 and Exo1 [9]–[11]. It loads at double-strand break sites and processively removes nucleotides from one DNA strand on each side of a break to render surrounding sequences progressively more single stranded. This single-stranded DNA is viewed as an obligate intermediate and is often used to define which recipient sequences are competent to search for potential donor sequences. Based on this model, extended resection at double-strand breaks is potentially dangerous to genome stability since it increases the likelihood that repetitive DNA in the vicinity of a break site will become single stranded and thus active as a recipient.

Indeed, we recently discovered in *Saccharomyces cerevisiae* that a double-strand break in unique DNA frequently activates as recipients repetitive DNA (Ty retrotransposons) up to 48 kb away from a break site, leading to non-allelic homologous recombination and a broad spectrum of genome rearrangements [12]. This break-distal recombination reveals that repeat sequences far from a break site are frequently activated and suggests that extensive resection occurs at break sites. Such extensive resection is supported by physical studies of double-strand breaks [7]. Furthermore, this activation of break-distal recipient repeats occurs even when break-proximal sequences have much more sequence identity with potential donors [12]. Thus, extensive resection appears to occur even when break-proximal sequences are competent for repair.

Given the danger of extended resection to genome stability, why does so much resection occur? If limited resection were sufficient for recombination, then in principle a double-strand break in unique sequences would activate only neighboring unique sequences as recipients. These unique-sequence recipients would constrain potential donors to the allelic region of the sister chromatid or homolog and thus bias towards non-mutagenic repair. Indeed, when resection is limited to only 0.1–1.0 kb of single-stranded DNA at break sites by inactivation of Sgs1 and Exo1, efficient homologous recombination repair ensues [10], [11]. However, whether limited resection stabilizes the genome is unclear. In fact, one study suggests that removing both Sgs1 and Exo1 can increase the likelihood that sequences near the break site undergo mutagenic break-induced replication at the expense of conservative gene conversion [13]. This observation led us to ask whether limited resection also increases the likelihood of mutagenic break-distal recombination.

In this study, we directly test the model that limited resection immediately around a double-strand break will suppress genome instability due to non-allelic homologous recombination between repetitive DNA sequences distal from the break site. We compare break-distal recombination between Ty repeat sequences in *S. cerevisiae* with and without Sgs1 and Exo1 resection machinery. Surprisingly, when resection is limited, rather than observing the expected decrease in break-distal recombination, we find a 200% increase in break-distal recombination and genome rearrangements. Limited resection therefore does not force recombination to use only sequences immediately surrounding the break site, but still allows break-distal recipient repeats to be efficiently used in the absence of Sgs1 and Exo1. We propose that any dangers associated with extended resection are tolerated to inhibit genome-destabilizing events such as break-distal recombination.

## Results

### Both Purebred and Hybrid Diploids Efficiently Repair Double-Strand Breaks in the Absence of Sgs1- and Exo1-Dependent Resection

To understand how limited resection impacts genome stability, we monitored double-strand break repair using a non-selective clone-based assay in diploid yeast that we previously developed [12]. In contrast to previous studies using assays that selected for a limited number of repair outcomes [13], [14], this genome-wide assay does not require selection of any specific repair outcome and allows us to measure the spectrum of repair products that results from a single double-strand break, including large rearrangements such as internal deletions, chromosome rings, and translocations [12]. This spectrum of rearrangements reflects a natural competition between various DNA substrates and repair processes that compete with one another during double-strand break repair.

**Figure 1:**
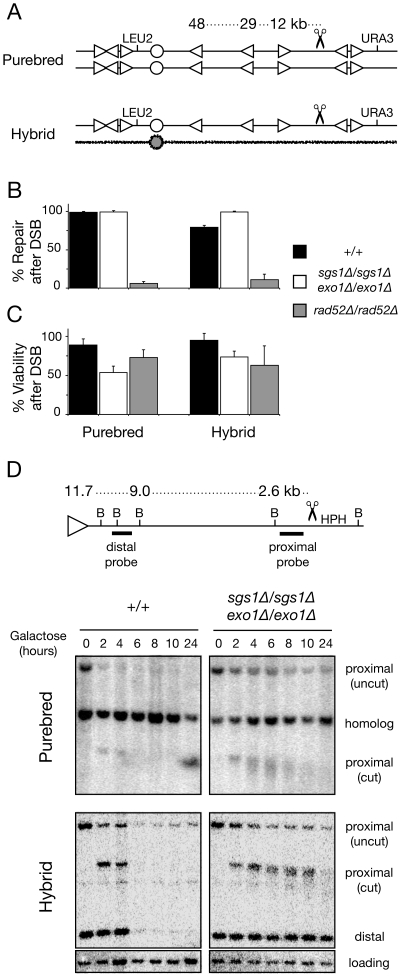
Majority of double-strand breaks in diploid genomes are repaired even without Sgs1- and Exo1-dependent long-range DNA resection. (A) Map of I-SceI cut site (scissors) on one *S. cerevisiae* chromosome III homolog in *S. cerevisiae/S. cerevisiae* purebred diploids (top) and *S. cerevisiae/S. bayanus* hybrid diploids (bottom), as previously described [12]. Heterozygous markers (*LEU2*, *URA3*) genetically monitor chromosome repair (Leu+ phenotype) or loss (Leu- Ura- phenotype) after an I-SceI-induced double-strand break. Three Ty retrotransposon elements (triangles) are located 12, 29, and 48 kilobases from the I-SceI cut site toward the centromere (circle) and may participate as break-distal recipient sequences for break-distal recombination [12]. Note that the *S. bayanus* genome is mostly devoid of Ty retrotransposon elements (no triangles on *S. bayanus* homeolog). (B) Frequencies of *S. cerevisiae* chromosome III repair (Leu+ phenotype) after I-SceI-induced double-strand break in purebred and hybrid diploid strains; wild-type purebred (MH3359), *sgs1Δ/sgs1Δ exo1Δ/exo1Δ* purebred (MH3736), *rad52Δ/rad52Δ* purebred (MH3475), wild-type hybrid (MH3360), *sgs1Δ/sgs1Δ exo1Δ/exo1Δ* hybrid (MH3747), *rad52Δ/rad52Δ* hybrid (MH3476). (C) Relative viability of wild-type, *sgs1Δ/sgs1Δ exo1Δ/exo1Δ*, and *rad52Δ/rad52Δ* purebred and hybrid diploids after I-SceI-induced double-strand breaks. Note that wild type and *rad52Δ/rad52Δ* data are reproduced from [12] for convenient reference. Error bars in B and C indicate standard deviation from at least three experiments. (D) Physical monitoring of DNA resection after galactose induction of I-SceI double-strand breaks using Southern blot analysis of BglII (B) digested genomic DNA in wild-type and *sgs1Δ/sgs1Δ exo1Δ/exo1Δ* purebred and hybrid diploids.

Briefly, in this assay we generate a single double-strand break at a precise location in diploid cells by integrating a copy of the recognition sequence for the I-SceI endonuclease onto *S. cerevisiae* chromosome III and expressing I-SceI protein from a galactose inducible promoter (Figure 1a). After plating cells at low density on non-selective media, each colony that grows represents an independent event that can be studied using a variety of genetic and physical assays to determine first whether repair occurred and second how repair occurred. Successful repair of a double-strand break can be detected by genotyping for the presence of the *LEU2* auxotrophic marker located near the chromosome III centromere (Figure 1a and Figure S1). Homologous recombination mediates most repair as repetitive sequences lie at breakpoint junctions and repair depends upon *RAD52* (Figure 1b). The use of diploids allows recovery of repair events that would otherwise be lethal in haploids due to loss of essential genetic material (Figure 1c). Because this assay does not select for any specific recombination product, we can monitor homologous recombination between natural repeats both near and far from a double-strand break. Furthermore, we can model hemizygotic genome regions where break-distal recombination is the primary repair pathway by creating a double-strand break in unique sequences far from repeats in hybrid diploids where allelic recombination occurs inefficiently due to low sequence identity (∼70%) between *S. cerevisiae* and *S.bayanus*.

To test whether break-distal recipient repeats can still recombine without extended resection, we deleted both copies of *SGS1* and *EXO1* in purebred and hybrid diploids and then verified the drug sensitivity phenotype (Figure S2). These diploids have an I-SceI cut site sequence integrated on the right arm of one *S. cerevisiae* chromosome III homolog in an 18 kb stretch of unique DNA (Figure 1a). Based upon previous published experiments [10], [11], we expected efficient repair of this double-strand break in the purebred *sgs1Δ/sgs1Δ exo1Δ/exo1Δ* strain using donor sequence from the homolog. Chromosome repair results in retention of the left arm and a Leu+ phenotype. After inducing double-strand breaks in exponentially growing cultures of purebred *sgs1Δ/sgs1Δ exo1Δ/exo1Δ* cells, 99% of cells were Leu+ indicating that they had efficiently repaired the break (Figure 1b, 1c, and Figure S1). These results indicate that an efficient process exists to repair double-strand breaks in purebred cells lacking Sgs1 and Exo1, consistent with previous results [7], [10], [11], [13], [14].

**Figure 2:**
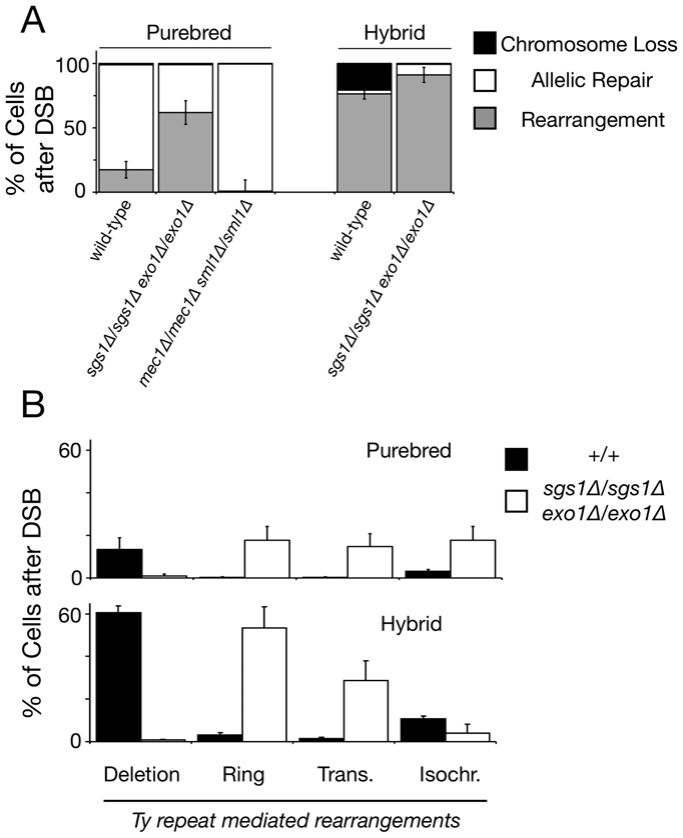
Recipient Ty elements located 12–48 kilobases away from the break site mediate chromosome rearrangements more frequently in *sgs1Δ/sgs1Δ exo1Δ/exo1Δ* mutant diploids than wild-type diploids. (A) Frequencies of outcomes after an I-SceI-induced double-strand break in purebred and hybrid diploids; wild-type purebred (MH3359), *sgs1Δ/sgs1Δ exo1Δ/exo1Δ* purebred (MH3736), *mec1Δ/mec1Δ sml1Δ/sml1Δ* purebred (FT5688), wild-type hybrid (MH3360), *sgs1Δ/sgs1Δ exo1Δ/exo1Δ* hybrid (MH3747). Repair colonies were analyzed as previously described [12] to determine whether repair occurred by allelic or non-allelic homologous recombination. Note that the relatively low frequency of rearrangements in *mec1Δ/mec1Δ sml1Δ/sml1Δ* mutants stems from a lack of internal deletions among our limited sampling of Ura+ clones (n = 10) and likely does not represent a significant decrease (0.0 observed vs. 1.6 predicted from wild type). (B) Frequencies of Ty-mediated rearrangements in wild type and *sgs1Δ/sgs1Δ exo1Δ/exo1Δ* mutants. Note that wild-type data was reproduced from [12] for convenient reference. Error bars represent standard error of the mean.

In contrast, we expected that the double-strand break in hybrid *sgs1Δ/sgs1Δ exo1Δ/exo1Δ* diploids should be poorly repaired by homologous recombination due to the low sequence identity (∼70%) between the homeologous sequences proximal to the break site. This poor repair would result in a higher frequency of chromosome loss, generating a Leu- phenotype. Instead, we observed very efficient repair as 99% of the cells surviving the break were Leu+ (Figure 1b, 1c, and S1). This efficient repair results in a 33-fold reduction in chromosome loss compared to wild-type hybrids (Figure 2a). To verify that long-range resection was compromised in these cells, we monitored single-stranded DNA formation at break sites using a standard physical assay. In this assay, resection is monitored by disappearance of a restriction fragment as resection destroys a flanking restriction site. Wild-type cells resected beyond a proximal 2.6 kb marker 4–6 hours after I-SceI expression, while this proximal region remained intact in *sgs1Δ/sgs1Δ exo1Δ/exo1Δ* mutants for at least 10 hours (Figure 1d). A distal 9.0 kb marker remained intact in hybrid diploid mutants even after 24 hours (Figure 1d). Thus, it appears unlikely that repetitive DNA 12–48 kb away from this break site is converted to single-stranded DNA via long-range resection even after 24 hours in *sgs1Δ/sgs1Δ exo1Δ/exo1Δ* mutants. Thus, an efficient repair process exists to repair double-strand breaks even in the hybrid strain lacking Sgs1 and Exo1 resection.

### Break-Distal Recombination Increases in the Absence of Sgs1 and Exo1

We were extremely surprised that hybrid diploids lacking Sgs1 and Exo1 could efficiently repair double-strand breaks in unique DNA as our physical analysis revealed that single-stranded DNA formation was limited to recipients that did not have a suitable donor in the genome. We therefore asked whether the efficient repair of double-strand breaks in *sgs1Δ/sgs1Δ exo1Δ/exo1Δ* cells occurred through break-distal or break-proximal sequences. As individual colonies may contain different populations of repaired chromosomes [7], [14], we first streaked each primary colony for single colonies and then characterized one secondary colony from each streak (discussed further below). We determined the structure of each repaired chromosome in these clones using a combination of pulsed-field gel electrophoresis, PCR fingerprinting, and array-comparative genome hybridization (Figure S3; Table S1). The sizes for most rearranged chromosomes are consistent with recombination between existing Ty elements. Indeed, Ty sequences map to the junctions of all rearrangements; these recipient Tys correspond to existing Ty elements 12–48 kb away from the break site. These rearrangements include translocations, chromosome rings, and deletions that we previously demonstrated occurred by break-distal recombination in wild-type cells [12].

These structural studies revealed that 91% (+/−6% s.e.m.) of *sgs1Δ/sgs1Δ exo1Δ/exo1Δ* hybrids and 62% (+/−9% s.e.m.) of *sgs1Δ/sgs1Δ exo1Δ/exo1Δ* purebreds suffering a double-strand break repaired the broken chromosome to give rise to genome rearrangements (Figure 2a). This represents a 20% and 250% increase in rearrangements relative to wild type for the hybrid and purebred respectively. These results have two surprising conclusions. First, break-distal recipient repeats can still be activated without Sgs1- and Exo1-dependent resection. Second, while repair of a double-strand break in purebreds is efficient, most repair is not with the homolog as would be expected if break-proximal sequences were exclusively used but rather with non-allelic donor repeats through break-distal recombination.

In addition to mutagenic break-distal recombination, short novel chromosome III rearrangements (<200 kb) were seen in 9% of the purebreds mutants and in 4% of the hybrids (Figure S3). These products likely result from *de novo* telomere addition, a process that occurs more frequently in cells lacking Sgs1 and Exo1 [7], [13], [14]. Finally, analyses in purebred *sgs1Δ/sgs1Δ exo1Δ/exo1Δ* diploids revealed that 87% of allelic repair occurred by mutagenic break-induced replication resulting in extensive loss of heterozygosity (Figures S1 and S3). Thus, insufficient resection activates mutagenic break-distal recombination and break-induced replication even in the presence of non-mutagenic repair pathways.

Importantly, the increase in break-distal recombination was not seen in *sgs1Δ/sgs1Δ* mutants [12; MH and DK, unpublished data] suggesting that this genome instability phenotype is not simply due to hyper-recombination associated with loss of Sgs1 [15]. This instability was also not seen in *msh6Δ/msh6Δ* mutants [12; MH and DK, unpublished data] suggesting that instability is not due solely to a failure to reject heteroduplexes [16]. Finally, *mec1Δ/mec1Δ* mutants did not show an increased level of rearrangements (Figure 2a and Figure S1) indicating that the genome instability associated with lack of Sgs1 and Exo1 is not simply due to an inability to activate a robust checkpoint response [9], [11]. Thus, increased break-distal recombination in cells lacking Sgs1 and Exo1 reveals a very efficient and previously unappreciated Sgs1- and Exo1-independent pathway to activate recipient repeat sequences very distal from a break site.

### Analysis of Genome Rearrangements Generated by Sgs1- and Exo1-Independent Break-Distal Recombination

To better understand how *sgs1Δ/sgs1Δ exo1Δ/exo1Δ* mutants repair double-strand breaks using break-distal recipient repeats, we subdivided the spectrum of genome rearrangements. Internal deletions between intrachromosomal Ty repeats flanking the break site occurred 10- to 80-fold less often in *sgs1Δ/sgs1Δ exo1Δ/exo1Δ* purebreds and hybrids compared to wild type (Figure 2b). This decrease is consistent with a lack of long-range resection, as these deletions likely form by single-strand annealing between direct Ty repeats flanking the break site that have been converted into single-stranded DNA in the same cell [12]. This finding lends further support that resection is limited in these mutants, consistent with previous studies [10], [11]. In contrast to internal deletions, 15% of purebred mutant cells and 29% of hybrids that suffered a double-strand break generated non-reciprocal translocations, a 20- to 50-fold increase in mutants compared to wild type (Figure 2b). These translocations may form by break-induced replication, which increases in the absence of Sgs1 or Exo1 [13], [14], or some currently unknown mechanism. Finally, 18% of purebred mutants and 53% of hybrids that suffered a double-strand break generated a chromosome ring, a 20- to 60-fold increase in mutants compared to wild type (Figure 2b). As chromosome rings are partially dependent upon *RAD51* [12], this finding suggests that at least some Rad51-mediated break-distal recombination events still occur without Sgs1 or Exo1.

To test directly the contribution of Rad51 to break-distal recombination when resection is limited, we deleted *RAD51* in purebred diploids lacking Sgs1 and Exo1. Repair efficiency remained high in *rad51Δ/rad51Δ sgs1Δ/sgs1Δ exo1Δ/exo1Δ* triple mutants, and most repaired chromosomes lost the centromere-distal *URA3* marker similar to *RAD51/RAD51 sgs1Δ/sgs1Δ exo1Δ/exo1Δ* double mutants (Figure S1). Examination of Ura− clones by pulsed-field gel electrophoresis revealed that 45% (10/22) of the Ura− clones in *rad51Δ/rad51Δ sgs1Δ/sgs1Δ exo1Δ/exo1Δ* triple mutants were small chromosome III products <200 kb (Figure S3e), 3-fold higher than the 15% (3/20) seen in *RAD51/RAD51 sgs1Δ/sgs1Δ exo1Δ/exo1Δ* double mutants (difference in proportions z = 2.13, p<0.05). The accumulation of these small chromosomes suggest that without Rad51, many broken chromosomes in *sgs1Δ/sgs1Δ exo1Δ/exo1Δ* mutants cannot be repaired and are instead capped by *de novo* telomere addition. Such increased *de novo* telomere addition has been previously observed in cells lacking Sgs1 and Exo1 [7], [13], [14]. Pulsed-field gel analysis revealed evidence of some chromosome rings and translocations (Figure S3e). That these rearrangements still occur without Rad51 can be explained by both the efficiency of Rad51-independent homologous recombination pathways in yeast [12], [17] and an inability of cells lacking Sgs1 and Exo1 to activate a robust cell cycle arrest (see below). Overall, these results suggest that Rad51 contributes at least partially to break-distal recombination in cells lacking Sgs1 and Exo1.

### Heterogeneous Population of Cells with Diverse Rearrangements Arise When Broken Chromosomes Are Propagated without Extended Resection

Even though a Mec1-checkpoint deficiency is not sufficient for increased break-distal recombination (Figure 2a), we wished to better understand how the lack of a robust cell cycle arrest affects repair of DNA damage [9], [11]. In cells lacking this cell cycle arrest checkpoint, both daughter cells could in principle inherit a broken chromosome. As each break is repaired independently, repair after cell division would generate genetically heterogeneous colonies. Consistent with this idea, previous studies of *sgs1Δexo1Δ* mutants found several colonies comprised of a mixture of cells that were repaired by either break-induced replication or *de novo* telomere addition [7], [14]. To determine whether break-distal recombination in *sgs1Δ/sgs1Δ exo1Δ/exo1Δ* mutants occurred before or after division, we examined whether colonies were homogeneous or heterogeneous for a given repaired chromosome.

**Figure 3:**
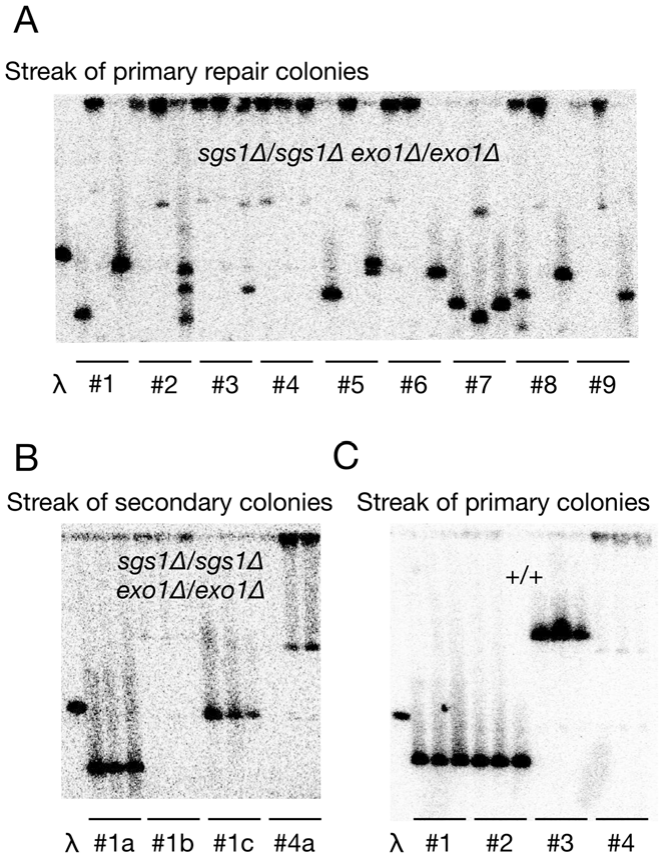
A single double-strand break on chromosome III generates descendants with different chromosome III structures in diploid cells lacking Sgs1 and Exo1. Physical analysis of *S. cerevisiae* chromosome III in hybrid diploids after repair of a I-SceI-induced double-strand break using pulsed-field gel electrophoresis (PFGE) followed by Southern blotting with a *LEU2* probe (parental size is 341 kb). (A) Nine primary repair colonies (#1 to #9) from *sgs1Δ/sgs1Δ exo1Δ/exo1Δ* parents were first streaked for single colonies and then three secondary colonies of this restreak were analyzed by PFGE. (B) A subset of secondary colonies shown in 3A were streaked for single colonies and another three colonies were analyzed by PFGE. (C) Primary repair colonies from wild-type parents were streaked for single colonies and three secondary colonies were analyzed. λ = PFGE ladder containing a 365 kb chromosome III (Bio-Rad #170-3605).

We struck out nine primary colonies for individual colonies from *sgs1Δ/sgs1Δ exo1Δ/exo1Δ* hybrid repair clones. From each of these primary streaks, we analyzed three secondary colonies by measuring the size of the repaired chromosome III by pulsed-field gel electrophoresis and Southern blotting against the left arm of chromosome III. As seen in Figure 3a, 8 out of 9 sets of secondary colonies contained multiple sizes. Half of these heterogeneous colonies showed three different sizes, suggesting that for these double-strand break events, at least two divisions occurred before all broken chromosomes arising from the original break were repaired. This heterogeneity was not simply due to inherent genome instability in *sgs1Δ/sgs1Δ exo1Δ/exo1Δ* mutants, as these repair products were stable upon additional streaking of these isolated clones (Figure 3b). Importantly, this heterogeneity was not observed with repair clones from wild-type cells (Figure 3c). These results suggest that Sgs1- and Exo1-dependent resection prevents double-strand breaks from producing a profusion of different genome rearrangements.

Since some mutant cells with a double-strand break divide at least two times before repairing all broken chromosomes, this situation increases the chance that a single double-strand break will give rise to at least one genome rearrangement. As there may be at least four chances to repair a double-strand break, this may explain why repair efficiency increases from 79% to 99% between wild-type hybrid and mutant cells (Figure 2a). This high repair efficiency in cells lacking Sgs1 and Exo1 also appears sufficient to rescue most of the repair defects in purebred diploids lacking Rad51. Specifically, *rad51Δ/rad51Δ* mutants repair broken chromosomes 70% of the time, while *rad51Δ/rad51Δ* mutants lacking Sgs1 and Exo1 repair broken chromosomes 96% of the time (Figure S1). Thus, lack of extensive resection appears to allow mutagenic substrates to persist providing increased opportunities to rearrange.

## Discussion

We previously showed that repetitive DNA sequences 12–48 kb distal from a double-strand break are activated as recipients and undergo homologous recombination with repeats elsewhere in the genome to generate genome rearrangements [12]. Based upon the literature the most likely mechanism to activate these repeats as recipients was extensive resection from the break by the Sgs1 and Exo1 resection machinery. However, we show here that when Sgs1- and Exo1-dependent resection is blocked in either hybrid or purebred yeast, rather than abolishing break-distal recombination, the majority of cells activate distal repeats and undergo break-distal recombination. Thus, this study provides evidence for a very efficient and previously unappreciated Sgs1- and Exo1-independent pathway that surprisingly activates recipient sequences very distal from the break site. That this pathway was not observed in previous studies of yeast cells lacking Sgs1 and Exo1 may be due to the fact that recombination reporters in these studies were biased against detection of break-distal recombination. Our findings reiterate the value of monitoring double-strand break repair in diploid yeast without selecting for specific outcomes using genome-wide assays [12].

How break-distal recipient repeats are activated in the absence of Sgs1 and Exo1 remains unclear but observations from this study and others provides important clues. This activation does not appear to be simply due to a failure to trigger a checkpoint response, as cells lacking the Mec1 checkpoint do not show an increased frequency of break-distal recombination (this study). Break-distal recipient repeats are unlikely to be activated through successive rounds of end processing by the Mre11/Rad50/Xrs2 complex. This process, which removes 50–100 bp at a time [10], would activate the 12 kb of unique sequences before activating the first break-proximal repeat and therefore should grossly favor repair using unique donor sequences on the homolog. The fact that repair by this alternative pathway often happens during subsequent cell cycles suggests that activation of distal repeats involves DNA replication in subsequent cell cycles possibly by inducing secondary double strand breaks [18], template switching [19], or unwinding of double-stranded DNA by a DNA helicase [20].

**Figure 4:**
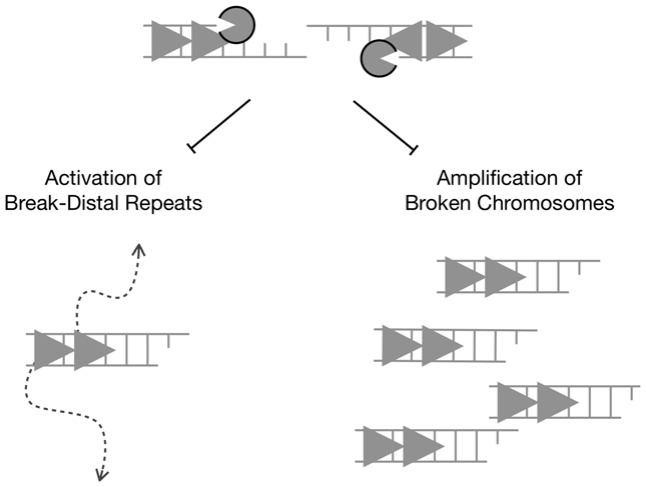
Long-range DNA resection at chromosome breaks promotes genome stability by constraining non-allelic homologous recombination between natural repeats. Summary of two key results in this study: Sgs1- and Exo1-dependent long-range resection at DNA double-strand breaks (left side) inhibits activation of break-distal repeats which in turn suppresses genome rearrangements and (right side) prevents the persistence of broken chromosomes that would otherwise be amplified by replication in subsequent cell cycles leading to a profusion of rearrangements in daughter cells.

This alternative resection-independent pathway that activates break-distal repeats is inhibited by resection as break-distal recombination occurs 3-fold less often in cells that resect (wild type) versus cells that do not resect (*sgs1Δ/sgs1Δ exo1Δ/exo1Δ*) (this study). Therefore, the idea that limited resection of double-strand breaks in non-repetitive DNA would promote genome stability by forcing recombination to use only sequences immediately surrounding the break site is incorrect. Rather, we find that the majority of cells lacking Sgs1 and Exo1 generate rearrangements using recipient repeats up to 48 kb away from the break site for non-allelic homologous recombination. Our finding reveals that an important biological function of resection is to inhibit genome instability by constraining recipient activation of repetitive DNA far from break sites (Figure 4). This extends and complements the importance of resection in constraining activation of repetitive DNA immediately at break sites [14].

We note that a previous study did not find increased break-distal recombination upon Sgs1 and Exo1 deletion when a double-strand break was induced at the mating type locus in a strain disomic for chromosome III ([7]). This difference may be due to the fact that the Ty repeats closest to the mating type locus (YCRCTy1–4 and YCRWTy1–5) are 33 kb away (vs 12–48 kb in our study). Alternatively, this difference may be due to the fact that repeats in an inverted orientation engage in different repair pathways ([21]).

We also show that cells lacking Sgs1 and Exo1 can generate multiple rearrangements from a single initial double-strand break. This amplification of DNA damage is due to the fact that Sgs1- and Exo1-dependent resection is required to degrade chromosomes, activate the DNA damage checkpoint, and arrest the cell cycle [9]–[11]. Without Sgs1 and Exo1, double-strand breaks can create stable chromosome fragments that replicate and propagate into subsequent daughter cells (Figure 4). As each broken chromosome is repaired independently with an increased likelihood for mutagenic break-distal recombination, this situation allows a single cell with a single double-strand break to give rise to a heterogeneous population of cells with diverse rearrangements. We note that this genomic heterogeneity after expansion of a single cell is reminiscent of cancer. Indeed, mutations in components of the mammalian DNA resection machinery contribute to tumorigenesis [22], [23]. Finally, we also note that limited resection could result when double-strand breaks form in genomic regions that are difficult to resect; at least one such loci has been previously described [11]. It may be enlightening to examine whether rearrangement hotspots are also regions of limited resection.

## Materials and Methods

### Yeast Strains and Recombination Assays

Strains are derived from S288C and were constructed as previously described [12]. *sgs1Δ/sgs1Δ exo1Δ/exo1Δ* mutants were created as both a purebred diploid (MH3736) and hybrid diploid (MH3747); *rad51Δ/rad51 sgs1Δ/sgs1Δ exo1Δ/exo1Δ* and *mec1Δ/mec1Δ sml1Δ/sml1Δ* mutants were created as purebred diploids (MH3798 and FT5688, respectively). Strains lacking Sgs1, Exo1, and Mec1 were phenotypically verified by drug sensitivities (Figure S2). We note that over the course of experiments, MH3747 was found to contain a pre-existing non-reciprocal translocation at the rDNA locus that exchanges the right arm of *S. cerevisiae* chromosome XII for the right arm of *S. bayanus* chromosome XII. This rearrangement does not appear to significantly affect double-strand break repair, as results are consistent between both diploid strains and with previous observations [7], [14].

Assays to determine viability, repair efficiency, and chromosome III structure after a double-strand break were previously described [12]. Experiments with wild type, *rad52Δ/rad52Δ*, and *sgs1Δ/sgs1Δ exo1Δ/exo1Δ* mutants were conducted concurrently but published separately [this study; 12]. Data for wild type and *rad52Δ/rad52Δ* mutants are reproduced in Figure 1b, 1c, and Figure 2 for reference.

### Physical Monitoring of DNA Resection

Purified genomic DNA was digested with BglII and separated on a 0.7% agarose gel. After transfer to nylon membranes, chromosomal fragments were monitored using radiolabeled probes. Purebred diploids were monitored using a probe against *SLM5*; hybrid diploids were monitored using probes against *SLM5*, *MAK32*, and *SPT2* (loading control).

## Supporting Information

Figure S1 Frequencies of genetic phenotypes for purebred and hybrid diploids after an I-SceI induced double-strand break. Phenotypes were determined by replica plating primary colonies from YPD agar plates to SC –leu and SC –ura agar plates as previously described [12]. *LEU2* lies on the left arm of chromosome III near the centromere; *URA3* lies on the right arm of chromosome III at the *BUD5* locus.
(TIF)

Figure S2 Verification of mutant strains by drug sensitivity. (a) Phleomycin plates were incubated for 3 days at 30°C for *S. cerevisiae* and 5 days at 23°C for *S. bayanus*. Strains are as follow: *S. cerevisiae MATα* wild-type (MH3356), *exo1Δ* (MH3708), *sgs1Δ* (MH3429), *sgs1Δexo1Δ* (MH3729); for *S. cerevisiae MATa* wild-type (MH3330), *exo1Δ* (MH3707), *sgs1Δ* (MH3423), sgs1Δ*exo1Δ* (MH3728); *S. bayanus MATa* wild-type (MH3399), *exo1Δ* (MH3744), *sgs1Δ* (MH3428), *sgs1Δexo1Δ* (MH3739). The *sgs1Δexo1Δ* haploid strains were mated to generate the diploid mutants used in this study. (b) MMS and HU plates were incubated for 3 days at 23°C. Strains are as follow: *S. cerevisiae MATα* wild-type (MH3356), *sml1Δ* (FT5679), *mec1Δsml1Δ* (FT5682); for *S. cerevisiae MATa* wild-type (MH3330), *sml1Δ* (FT5678), *mec1Δsml1Δ* (FT5681). The *mec1Δsml1Δ* haploid strains were mated to generate the diploid mutants used in this study.
(TIF)

Figure S3 Molecular analysis of repair clones from diploids lacking Sgs1 and Exo1. (a and b) PFGE/Southern analysis of repair clones from purebred diploids lacking Sgs1 and Exo1. (c) PCR fingerprinting of hybrid mutant repair clones. Coordinates of each locus examined are specified in Table S1. (d) PFGE/Southern analysis of repair clones from hybrid diploids lacking Sgs1 and Exo1. Repeated analysis of R190, R191, and R328 revealed single bands at the indicated sizes. (e) PFGE/Southern analysis of repair clones from purebred diploids lacking Rad51, Sgs1, and Exo1. All Southern blots used a *LEU2* probe; parental size of chromosome III is 341 kb.
(TIF)

Table S1 Summary of data used to classify repair clones. ^a^PCR fingerprinting provides information on which recipient was used by indicating presence or absence of a particular *S. cerevisiae* chromosome III segment that lies immediately CEN-proximal to a given chromosome element. “A” is adjacent to YCRCdelta6 (123535 nt–123698 nt), “B” is adjacent to YCRCdelta7 (142402 nt–142635 nt), “C” is adjacent to YCRWTy1-2/1-3 (148580 nt – a position 497 nt centromere-distal that is not annotated in SGD (see [12])), and “D” is adjacent to the I-SceI cutsite (153187 nt–153887 nt).
(DOC)
