# Supplementary figures and images for: DNA Resection at Chromosome Breaks Promotes Genome Stability by Constraining Non-Allelic Homologous Recombination
Source: PLoS Genet. 2012 Mar 29;8(3):e1002633. doi: 10.1371/journal.pgen.1002633 (PMC3315486; doi:10.1371/journal.pgen.1002633)

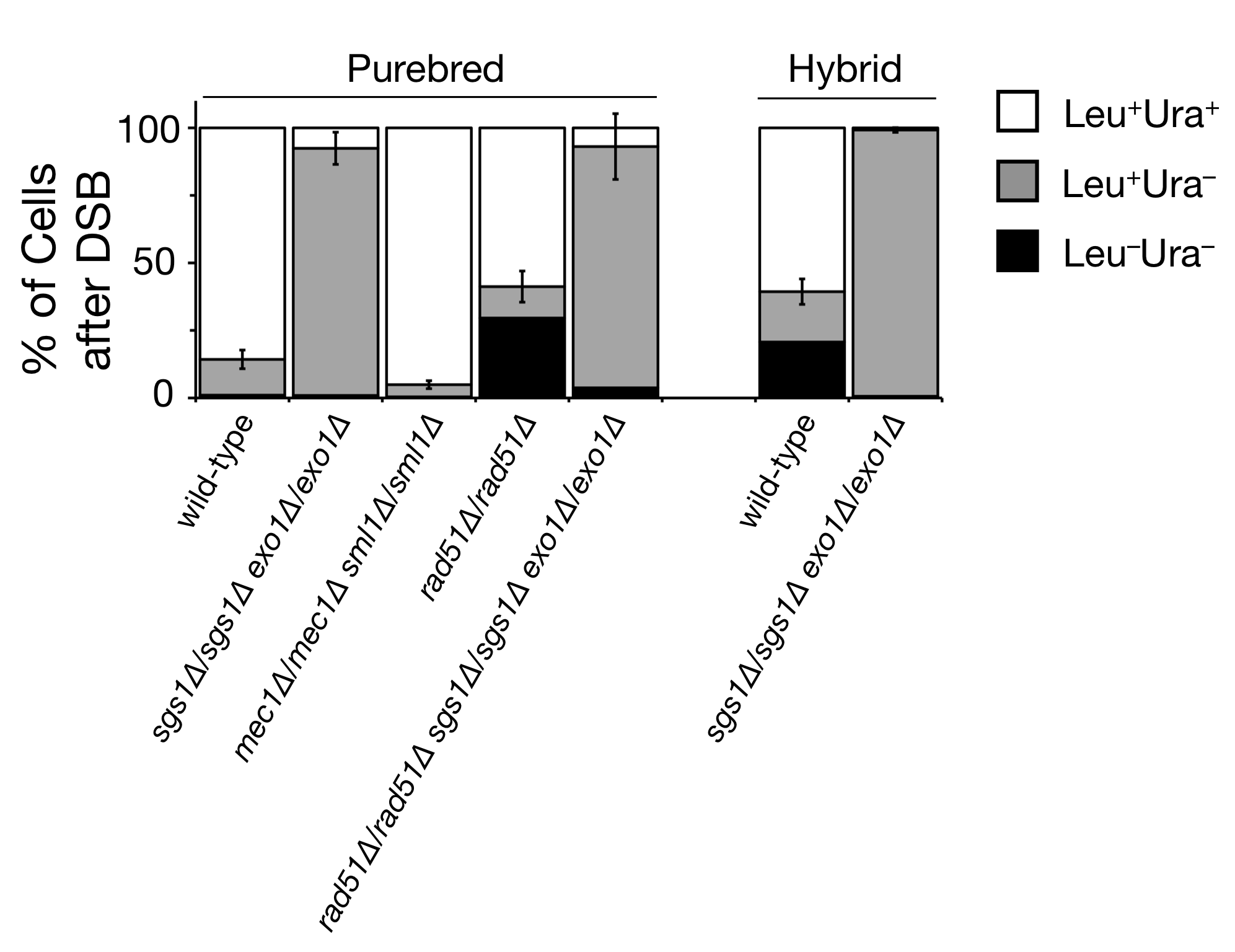

Supplement: Figure S1 — Frequencies of genetic phenotypes for purebred and hybrid diploids after an I-SceI induced double-strand break. Phenotypes were determined by replica plating primary colonies from YPD agar plates to SC –leu and SC –ura agar plates as previously described [12]. LEU2 lies on the left arm of chromosome III near the centromere; URA3 lies on the right arm of chromosome III at the BUD5 locus. (TIF) [file pgen.1002633.s001.tif]

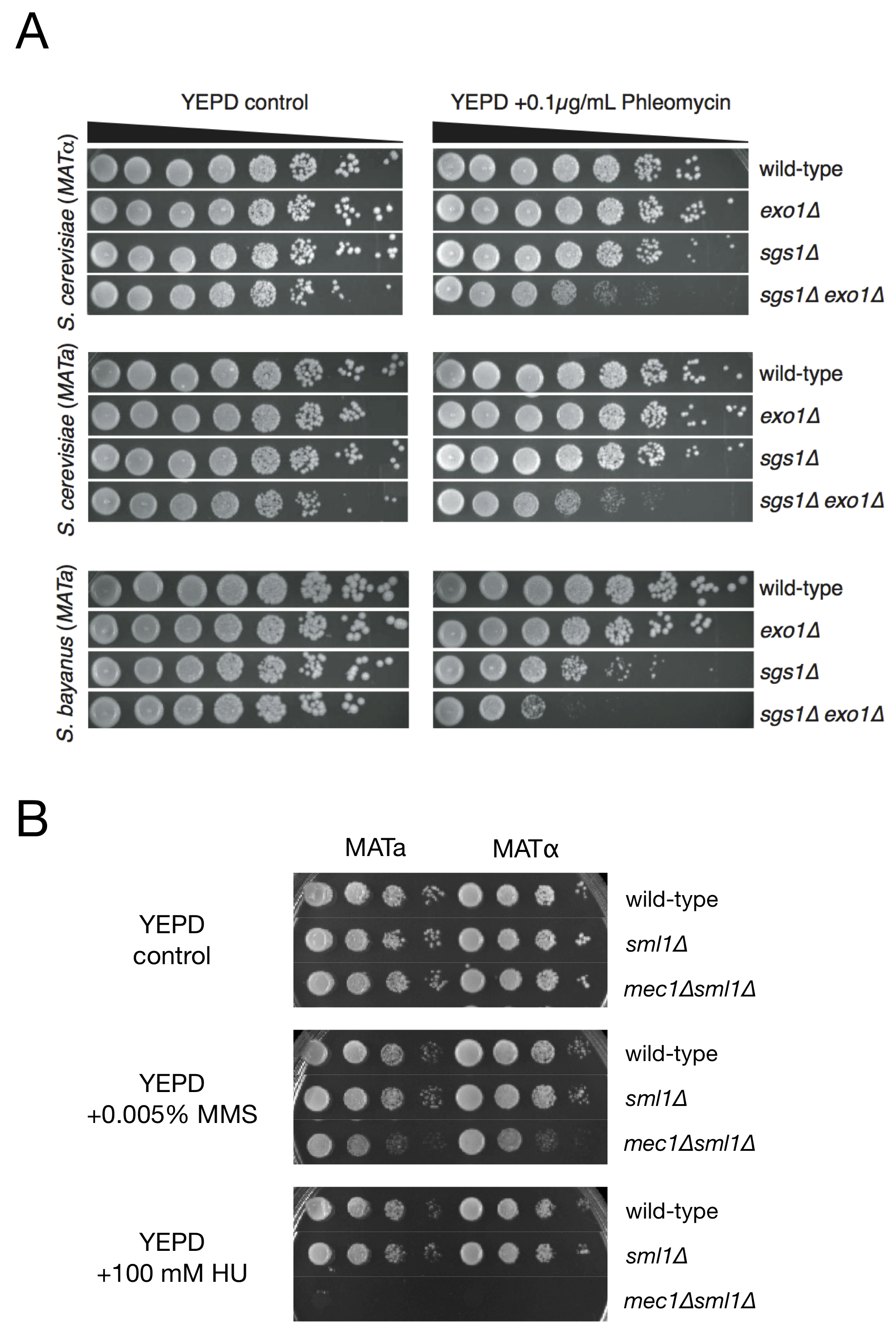

Supplement: Figure S2 — Verification of mutant strains by drug sensitivity. (a) Phleomycin plates were incubated for 3 days at 30°C for S. cerevisiae and 5 days at 23°C for S. bayanus. Strains are as follow: S. cerevisiae MATα wild-type (MH3356), exo1Δ (MH3708), sgs1Δ (MH3429), sgs1Δexo1Δ (MH3729); for S. cerevisiae MATa wild-type (MH3330), exo1Δ (MH3707), sgs1Δ (MH3423), sgs1Δexo1Δ (MH3728); S. bayanus MATa wild-type (MH3399), exo1Δ (MH3744), sgs1Δ (MH3428), sgs1Δexo1Δ (MH3739). The sgs1Δexo1Δ haploid strains were mated to generate the diploid mutants used in this study. (b) MMS and HU plates were incubated for 3 days at 23°C. Strains are as follow: S. cerevisiae MATα wild-type (MH3356), sml1Δ (FT5679), mec1Δsml1Δ (FT5682); for S. cerevisiae MATa wild-type (MH3330), sml1Δ (FT5678), mec1Δsml1Δ (FT5681). The mec1Δsml1Δ haploid strains were mated to generate the diploid mutants used in this study. (TIF) [file pgen.1002633.s002.tif]

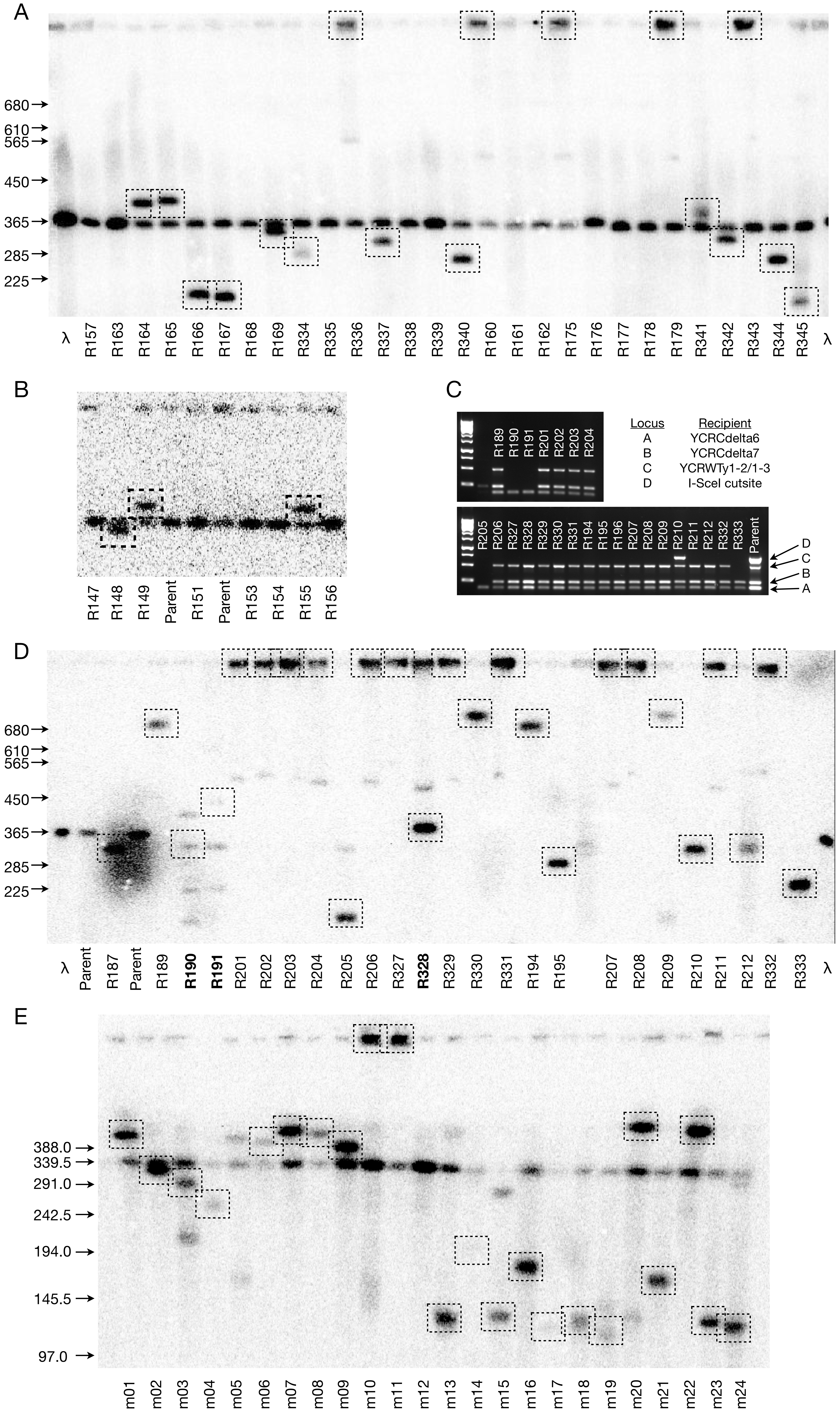

Supplement: Figure S3 — Molecular analysis of repair clones from diploids lacking Sgs1 and Exo1. (a and b) PFGE/Southern analysis of repair clones from purebred diploids lacking Sgs1 and Exo1. (c) PCR fingerprinting of hybrid mutant repair clones. Coordinates of each locus examined are specified in Table S1. (d) PFGE/Southern analysis of repair clones from hybrid diploids lacking Sgs1 and Exo1. Repeated analysis of R190, R191, and R328 revealed single bands at the indicated sizes. (e) PFGE/Southern analysis of repair clones from purebred diploids lacking Rad51, Sgs1, and Exo1. All Southern blots used a LEU2 probe; parental size of chromosome III is 341 kb. (TIF) [file pgen.1002633.s003.tif]
