# Supplementary material for: DNA Resection at Chromosome Breaks Promotes Genome Stability by Constraining Non-Allelic Homologous Recombination
Source: PLoS Genet. 2012 Mar 29;8(3):e1002633. doi: 10.1371/journal.pgen.1002633 (PMC3315486; doi:10.1371/journal.pgen.1002633)
Supplement: Table S1 — Summary of data used to classify repair clones. aPCR fingerprinting provides information on which recipient was used by indicating presence or absence of a particular S. cerevisiae chromosome III segment that lies immediately CEN-proximal to a given chromosome element. “A” is adjacent to YCRCdelta6 (123535 nt–123698 nt), “B” is adjacent to YCRCdelta7 (142402 nt–142635 nt), “C” is adjacent to YCRWTy1-2/1-3 (148580 nt – a position 497 nt centromere-distal that is not annotated in SGD (see [12])), and “D” is adjacent to the I-SceI cutsite (153187 nt–153887 nt). (DOC) [file pgen.1002633.s004.doc]

**TABLE S1.** Summary of data used to classify repair clones

| **Purebred *sgs1∆/sgs1∆ exo1∆/exo1∆* Repair Clones (from MH3736)** | | | | | | | | |
| --- | --- | --- | --- | --- | --- | --- | --- | --- |
|  | Clone | Rearrangement | Recipient | Distance  from DSB | Phenotype | PFGE Size |  |  |
|  | R148 | Deletion | YCRWTy1-2/1-3 | 12 kb | Leu^+^Ura^+^ | 320 |  |  |
|  | R149 | Other |  |  | Leu^+^Ura^+^ | 375 |  |  |
|  | R155 | Other |  |  | Leu^+^Ura^+^ | 375 |  |  |
|  |  |  |  |  |  |  |  |  |
|  | R164 | Translocation |  |  | Leu^+^Ura^-^ | 400 |  |  |
|  | R165 | Translocation |  |  | Leu^+^Ura^-^ | 400 |  |  |
|  | R166 | Other |  |  | Leu^+^Ura^-^ | <200 |  |  |
|  | R167 | Other |  |  | Leu^+^Ura^-^ | <200 |  |  |
|  | R169 | Translocation |  |  | Leu^+^Ura^-^ | 320 |  |  |
|  | R334 | Isochromosome | YCRWTy1-2/1-3 | 12 kb | Leu^+^Ura^-^ | 270 |  |  |
|  | R336 | Ring | YCRWTy1-2/1-3 | 12 kb | Leu^+^Ura^-^ | well |  |  |
|  | R337 | Isochromosome | YCRWTy1-2/1-3 | 12 kb | Leu^+^Ura^-^ | 290 |  |  |
|  | R340 | Isochromosome | YCRWTy1-2/1-3 | 12 kb | Leu^+^Ura^-^ | 270 |  |  |
|  | R160 | Ring | YCRWTy1-2/1-3 | 12 kb | Leu^+^Ura^-^ | well |  |  |
|  | R175 | Ring | YCRWTy1-2/1-3 | 12 kb | Leu^+^Ura^-^ | well |  |  |
|  | R179 | Ring | YCRWTy1-2/1-3 | 12 kb | Leu^+^Ura^-^ | well |  |  |
|  | R341 | Translocation |  |  | Leu^+^Ura^-^ | 375 |  |  |
|  | R342 | Translocation |  |  | Leu^+^Ura^-^ | 300 |  |  |
|  | R343 | Ring | YCRWTy1-2/1-3 | 12 kb | Leu^+^Ura^-^ | well |  |  |
|  | R344 | Isochromosome | YCRWTy1-2/1-3 | 12 kb | Leu^+^Ura^-^ | 270 |  |  |
|  | R345 | Other |  |  | Leu^+^Ura^-^ | <200 |  |  |
|  | R612 | Isochromosome | YCRCdelta7 | 29 kb | Leu^+^Ura^-^ | 240 |  |  |
|  | R615 | Ring | YCRWTy1-2/1-3 | 12 kb | Leu^+^Ura^-^ | well |  |  |
|  | R625 | Isochromosome | YCRWTy1-2/1-3 | 12 kb | Leu^+^Ura^-^ | 285 |  |  |
|  |  |  |  |  |  |  |  |  |
|  |  |  |  |  |  |  |  |  |
| **Hyrbid *sgs1∆/sgs1∆ exo1∆/exo1∆* Repair Clones (from MH3747)** | | | | | | | | |
|  | Clone | Rearrangement | Recipient | Distance  from DSB | Phenotype | PFGE Size | PCR^a^ | aCGH Slide |
|  | R187 | Deletion | YCRWTy1-2/1-3 | 12 kb | Leu^+^Ura^+^ | 320 |  |  |
|  |  |  |  |  |  |  |  |  |
|  | R189 | Translocation | YCRWTy1-2/1-3 | 12 kb | Leu^+^Ura^-^ | >680 | ABC | 4765 |
|  | R190 | Allelic | *near YCR005C* | 50 kb | Leu^+^Ura^-^ | 340 | A | 4691 |
|  | R191 | Ring | YCRCdelta6 | 48 kb | Leu^+^Ura^-^ | 440 | A | 5086 |
|  | R201 | Ring | YCRWTy1-2/1-3 | 12 kb | Leu^+^Ura^-^ | well | ABC | 5133 |
|  | R202 | Ring | YCRWTy1-2/1-3 | 12 kb | Leu^+^Ura^-^ | well | ABC |  |
|  | R203 | Ring | YCRWTy1-2/1-3 | 12 kb | Leu^+^Ura^-^ | well | ABC |  |
|  | R204 | Ring | YCRWTy1-2/1-3 | 12 kb | Leu^+^Ura^-^ | well | ABC |  |
|  | R205 | Other |  |  | Leu^+^Ura^-^ | <200 | A |  |
|  | R206 | Ring | YCRWTy1-2/1-3 | 12 kb | Leu^+^Ura^-^ | well | ABC |  |
|  | R327 | Ring | YCRWTy1-2/1-3 | 12 kb | Leu^+^Ura^-^ | well | ABC |  |
|  | R328 | Translocation |  |  | Leu^+^Ura^-^ | 380 | ABC |  |
|  | R329 | Ring | YCRWTy1-2/1-3 | 12 kb | Leu^+^Ura^-^ | well | ABC |  |
|  | R330 | Translocation |  |  | Leu^+^Ura^-^ | >680 | ABC |  |
|  | R331 | Ring | YCRWTy1-2/1-3 | 12 kb | Leu^+^Ura^-^ | well | ABC |  |
|  | R194 | Translocation |  |  | Leu^+^Ura^-^ | >680 | ABC |  |
|  | R195 | Translocation |  |  | Leu^+^Ura^-^ | 300 | ABC |  |
|  | R207 | Ring | YCRWTy1-2/1-3 | 12 kb | Leu^+^Ura^-^ | well | ABC | 4887 |
|  | R208 | Ring | YCRWTy1-2/1-3 | 12 kb | Leu^+^Ura^-^ | well | ABC |  |
|  | R209 | Translocation |  |  | Leu^+^Ura^-^ | >680 | ABC |  |
|  | R210 | Allelic | *near YCR025C* | <1 kb | Leu^+^Ura^-^ | 340 | ABCD | 4889 |
|  | R211 | Ring | YCRWTy1-2/1-3 | 12 kb | Leu^+^Ura^-^ | well | ABC |  |
|  | R212 | Translocation |  |  | Leu^+^Ura^-^ | 340 | ABC | 4890 |
|  | R332 | Ring | YCRWTy1-2/1-3 | 12 kb | Leu^+^Ura^-^ | well | ABC |  |
|  | R333 | Isochromosome | YCRCdelta7 | 29 kb | Leu^+^Ura^-^ | 230 | AB | 4886 |

^a^PCR fingerprinting provides information on which recipient was used by indicating presence or absence of a particular *S. cerevisiae* chromosome III segment that lies immediately CEN-proximal to a given chromosome element. “A” is adjacent to YCRCdelta6 (123535 nt – 123698 nt), “B” is adjacent to YCRCdelta7 (142402 nt – 142635 nt), “C” is adjacent to YCRWTy1-2/1-3 (148580 nt – a position 497 nt centromere-distal that is not annotated in SGD (see Hoang, et al., 2010 PLoS Genetics)), and “D” is adjacent to the I-SceI cutsite (153187 nt – 153887 nt)
